# 

**Published:** 1937

**Authors:** 


### The Medical Library of the University of Bristol,

WITH WHICH IS INCORPORATED

**The Library of the Bristol Medico-Chirurgical Society.**

---

*The following donations have been received since the publication of the list in May,* 1937.

June, 1937.

| | | |
|---|---|---|
| American Laryngological Association (1) | .. | 1 volume. |
| American Laryngological, Rhinological and Otological Society (2) .. .. | .. | 1 ,, |
| American Otological Society (3) .. | .. | 1 ,, |
| Mrs. B. Howell Jones (4) .. .. | .. | 6 volumes. |
| Messrs. H. K. Lewis and Co. Ltd. (5) | .. | 1 volume. |
| Milk Nutrition Committee (6) .. .. | .. | 1 ,, |
| Otago University Medical School (7) .. | .. | 1 ,, |
| The late Dr. George Parker (8) .. | .. | 15 volumes. |
| Mr. E. Watson-Williams (9) .. .. | .. | 4 ,, |
| Dr. P. Watson-Williams (10) .. .. | .. | 1 pamphlet |

Unbound periodicals have also been received from Professor E. W. Hey Groves, Professor C. Bruce Perry, Dr. J. Odery Symes and Dr. T. Milling.

### THE ONE HUNDRED AND FIFTY-NINTH LIST OF BOOKS.

The figures in round brackets refer to the figures after the names of the donors. The books to which no such figures have been attached have either been bought from the Library Fund or received through the *Journal*.

| | | | |
|---|---|---|---|
| **Allen, F. M. B.** .. | *Aids to the Treatment of Diseases of Children* | 7th Ed. | 1937 |
| **Bailey, Hamilton** .. | *Clinical Surgery for Dental Practitioners* | (5) | 1937 |
| **Bailey, Hamilton** .. | *Diseases of the Testicle* .. .. .. .. .. | | 1936 |
| **Blacker, C. P.** .. .. | *A Social Problem Group?* .. .. .. .. | | 1937 |
| **Boerhaave, H.** .. .. | *Medical Correspondence* .. .. .. .. | (8) | 1745 |

| | | |
|---|---|---|
| **Boerhaave, H.** | *A Method of Studying Physick.* Trans. Samber (8) | 1719 |
| **Budd, George** | *On Diseases of the Liver* 2nd Ed. (4) | 1852 |
| **Carpenter, Wm. B.** | *Principles of Human Physiology* 4th Ed. (4) | 1853 |
| **Castle, W. B., and Minot, C. R.** | *Pathological Physiology and Clinical Description of the Anœmias* | 1936 |
| *Charterhouse Rheumatism Clinic.* | *Original Papers.* Vol. I | 1937 |
| **Clowes, F., and Coleman, J. B.** | *Quantitative Chemical Analysis* 8th Ed. (9) | 1909 |
| **Cooke, J.** | *Supplementum Chirurgiœ* (8) | 1655 |
| **Coombs, C. F.** | *Rheumatic Heart Disease* (8) | 1924 |
| **Descour, L.** | *Pasteur and his Work.* Trans. by Wedd (8) | 1922 |
| **Dickinson, K. Shallcross** | *The Main Points of Chemical Warfare from the Medical Aspect* | 1937 |
| **Evans, Griffith** | *Latent Syphilis and the Autonomic Nerve System* 2nd Ed. | 1937 |
| **Gray, H.** | *Anatomy, Descriptive and Surgical* 7th Ed. (8) | 1875 |
| **Gray, H.** | *Anatomy, Surgical and Practical* 11th Ed. (9) | 1887 |
| **Hammond, T. E.** | *Infections of the Urinary Tract* | 1935 |
| **Harris, I.** | *High Blood Pressure* | 1937 |
| **Harris, Wilfred** | *The Facial Neuralgias* | 1937 |
| **Hopewell-Ash, Edwin** | *Diagnosis of some Delusional Insanity Types in General Practice* | 1936 |
| **Hutchison, Robert** | *Principles of Diagnosis, Prognosis and Treatment* 2nd Ed. | 1937 |
| **Jenner, Edward** | *The Notebook of Edward Jenner* (8) | 1931 |
| **Le Clerc** | *The Compleat Surgeon* 6th Ed. (8) | 1725 |
| **Le Fleming, E. Kaye** | *An Introduction to General Practice* | 1936 |
| **Lyle, K. and Jackson, S.** | *Practical Orthoptics in the Treatment of Squint* | 1937 |
| **MacMichael, W.** | *The Gold-Headed Cane* 2nd Ed. (8) | 1828 |
| **Mead, Richard** | *A Short Discourse concerning Pestilential Contagion* (8) | 1722 |
| *Milk and Nutrition.* | Part I (6) | 1937 |
| **Miller, James** | *Principles of Surgery.* 2nd and 3rd Eds. (4) | 1852–3 |
| *Ministry of Health* | *Advisory Committee on Nutrition.* First Report | 1937 |
| **Morton, Dudley J.** | *The Human Foot* | 1935 |
| **Osman, A. Arnold** | *Original Papers of Richard Bright on Renal Disease* | 1937 |
| **Paterson, Donald** | *Sick Children, Diagnosis and Treatment* 2nd Ed. | 1937 |
| **Prout, William** | *On the Nature and Treatment of Stomach and Renal Diseases* 5th Ed. (4) | 1848 |
| **Quain, Jones** | *Elements of Anatomy.* Vol. II 5th Ed. (4) | 1845 |
| **Roche, A. E.** | *Urology in General Practice* | 1938 |
| **Sachs, Wulf** | *The Vegetative Nervous System* | 1936 |
| **Schwarz, G., Goldberger, J. and Crocker, C.** | *Diseases of the Colon and Rectum* | 1937 |
| **Sears, W. Gordon** | *Vade Mecum of Medical Treatment* | 1937 |
| **Semon, Felix** | *Forschungen und Erfahrungen,* 1880–1910. 2 Vols. (9) | 1912 |

| | | | |
|---|---|---|---|
| **Smart, Morton** .. .. | *Graduated Muscular Contractions* .. .. .. .. | | 1936 |
| **Smith, N. Ross** .. | *Elements of Orthopædic Surgery* .. .. .. | | 1937 |
| **Snell, Sidney H.** .. | *A Doctor at Work and Play* .. .. .. | | 1937 |
| **Sprigge, S. S.** .. .. | *Physic and Fiction* .. .. .. .. .. .. .. | (8) | 1921 |
| **Stockman, R.** .. .. | *Rheumatism and Arthritis* .. .. .. | (8) | 1921 |
| **Thornton, R. J.** .. | *A Family Herbal* .. .. .. .. .. .. | (8) | 1814 |
| **Vaughan, Kathleen** .. | *Safe Childbirth* .. .. .. .. .. .. .. .. | | 1937 |
| **Watson-Williams, P.**.. | *Tecnica personale di explorazione dei sini paranasali* .. .. .. .. .. .. .. | (10) | 1937 |
| **Whitwell, J. R.**.. .. | *Historical Notes on Psychiatry* .. .. .. | | 1936 |
| **Woodall, J.** .. .. | *The Surgeon's Mate* .. .. .. .. | (8) | 1639 (?) |
| **Zimmern, A. and Perol, P.** | *Electro-Diagnosis in War* .. .. .. | (8) | 1918 |

### TRANSACTIONS, REPORTS, JOURNALS, ETC.

| | | |
|---|---|---|
| Medical Annual, 1937 .. .. .. .. .. .. .. .. .. .. .. .. .. .. | | 1937 |
| Proceedings of the University of Otago Medical School. No. 14 | (7) | 1937 |
| Quarterly Cumulative Index Medicus. Vol. 20 (1936).. .. .. .. .. | | 1937 |
| St. Thomas' Hospital Reports. Vol. LVIII (1934) .. .. .. .. .. | | 1936 |
| Transactions of the Fifty-eighth Annual Meeting of the American Laryngological Association.. .. .. .. .. .. .. .. .. .. .. | (1) | 1936 |
| Transactions of the American Laryngological, Rhinological and Otological Society. Forty-second Meeting .. .. .. .. .. | (2) | 1936 |
| Transactions of the American Otological Society. Vol. XXVI | (3) | 1936 |

### Publications Received

From EDWARD ARNOLD & Co. :

*The Common Neuroses.* By T. A. ROSS, M.D., F.R.C.P. 2nd Edition. Price 10s. 6d.

From JOHN BALE, SONS & DANIELSSON LTD. :

*Failure of the Heart and Circulation.* By TERENCE EAST, M.A., D.M., F.R.C.P. Price 2s. 6d.

From DR. P. BARON :

*Conduite à tenir en presence d'une colibacillose urinaire chronique.* By Dr. PAUL BARON. Price 5 francs.

From J. M. DENT & SONS LTD. :

*What is Osteopathy?* By CHARLES HILL, M.A., M.D., D.P.H., and H. A. CLEGG, M.A., M.B., M.R.C.P. Price 7s. 6d.

From WILLIAM HEINEMANN LTD. :

*Clinical Contraception.* By GLADYS M. COX, M.B., B.S. 2nd Ed. Price 7s. 6d.

From H. K. LEWIS & CO. LTD. :

*Milk Products.* By W. CLUNIE HARVEY and H. HILL. Price 6s.

*Common Skin Diseases.* By A. C. ROXBURGH, M.A., M.D., B.Ch., F.R.C.P. 4th Edition. Price 15s.

*The Morphine Habit.* By G. LAUGHTON SCOTT, M.R.C.S., B.A. 2nd Edition. Price 5s.

From E. & S. LIVINGSTONE :

*Botany.* Part II. (Catechism Series.) 4th Edition. Price 1s. 6d.

*Elementary Genetics.* By HANS GRÜNEBERG, Ph.D., M.D. Price 1s. 6d.

From OXFORD UNIVERSITY PRESS :

*Chronic Miliary Tuberculosis.* By CLIFFORD HOYLE, M.D., M.R.C.P., and MICHAEL VAIZEY, M.B., M.R.C.P. Price 12s. 6d.

*The Control of Tuberculosis in England.* By G. GREGORY KAYNE, M.D., M.R.C.P., D.P.H. Price 8s. 6d.

*Diseases of the New Born.* By ABRAHAM TOW, M.D. Price 27s. 6d.

*Tweedy's Practical Obstetrics.* 7th Edition by SOLOMONS and FALKINER. Price 25s.

From JOHN WRIGHT & SONS LTD. :

*British Journal of Surgery.* Vol. XXIV. No. 96. (April, 1937.) Price 12s. 6d.

### Local Medical Notes

EXAMINATION RESULTS.

**University of Bristol.**—Students of the University have recently passed the following examinations :—

M.B., Ch.B.—*First Examination. Pass :* Doris E. Counsell, Moragh J. Dickson, Mary D. Dixon, Elizabeth Hodson, A. G. H. Mitchinson, J. L. Pardoe, Margaret Savile, I. F. R. Sutherland, G. H. Valentine, R. F. Brooks, J. Deighton, J. Jones, N. Slade. *Second Examination* (*Section I*). *Pass :* K. L. Brown, J. T. N. Cole, D. I. Fullerton, F. J. Goddard, P. Legat, Madeleine I. Finzel, T. E. Lloyd, H. Mullen, R. W. Orton. *Final Examination* (*Section I*). *Pass :* Sybil M. Williams. (*Section II*). *Pass :* P. B. Ryan (*with Second Class Honours*), Daphne V. Dennis, J. P. M. Forde, C. R. G. Howard, N. R. Matheson, A. N. H. Peach (with Distinction in Surgery), J. W. E. Snawdon, A. M. Spencer, R. Tallack, P. Zimmering, H. James, Aldyth D. Jones, P. N. Heron, P. K. Jenkins.

B.D.S.—*First Examination. Pass:* F. C. Bryant, P. B. Carey, G. Hellings, G. K. G. Jennings, Sarah Jones, P. M. Nicholas. *Second Examination. Pass:* F. C. Baker. *Third Examination. Pass:* R. J. Gething, G. H. Bell, J. E. H. Bell, J. F. Blenkinsop, J. R. D. Gordon, A. A. Grant (with Distinction), G. R. Styles.

L.D.S.—*First Examination. Pass:* W. D. Arnold, G. J. Mills. *Second Examination. Pass:* D. G. Davies, J. R. Herbert, J. W. S. Jenkins, C. R. Sage, J. C. Jowett, R. B. Stiles. *Third Examination. Pass:* J. F. Durie, L. K. A. S. Oxley, Peggy Rooke, H. B. Harding, D. M. Sanders, L. H. Barker-Tufft, F. W. Clift, K. A. Gordon-Ralph, G. H. Hudson, E. L. Manton, R. H. G. Sims, Mary E. Thomas, May A. Trump, L. N. Weale, E. S. Wolfson. *Final Examination. Pass:* M. G. Davies, J. B. Inverdale, P. H. S. Paine, H. W. Williams, J. G. Windmill.

*Midwife Teachers' Certificate Examination (Part II). Pass:* Annie A. Barnes, Frances E. L. M. Harper, Dorothy Humphreys.

**Conjoint Board.** — M.R.C.S., L.R.C.P. — *Pass: In Pharmacology and Materia Medica):* R. T. Moore. *In Medicine:* E. J. Lace. *In Surgery:* Mabel W. N. Tribe, R. L. J. S. Derham, J. H. Bulleid. *In Pathology and Midwifery:* P. W. J. Parkes. *In Pathology:* Rotha H. Dalton.

L.D.S., R.C.S. — *Pass: In Special Anatomy and Physiology:* S. G. Sheffield. *Final Examination (Part II). Pass:* J. R. P. Thomas.

**Royal College of Surgeons.**—F.R.C.S.—*Primary:* S. D. Loxton.

---

APPOINTMENTS.

**B.R.I. and B.G.H., Radiological Department.**—Drs. F. G. Bergin, G. B. Bush and S. B. Adams.

---

The Long Fox Memorial Lecture will be delivered in the University on Tuesday, 19th October, 1937, at 8.30 p.m., by Professor V. B. Green-Armytage, M.D., F.R.C.P., F.C.O.G. Subject: **"The Debt of Western Medicine to the East."**

**Bristol Royal Infirmary Bicentenary.**—The arrangements for the celebration of this anniversary are now well in hand. The Lord Mayor has generously offered to hold a civic reception in the evening of 21st October, and there will be a dinner, and possibly a dance, on 22nd October. It is hoped these will afford opportunities to all classes interested in the Infirmary—Patrons, Governors, Staff, and past and present Students and Nurses. We would particularly ask former Students to remember the date. Further particulars will be published later, or can be obtained from the Honorary Secretary of the Bicentenary Celebrations Committee, Mr. E. Watson-Williams, 65 Pembroke Road, Clifton, Bristol 8.

---

**Royal College of Surgeons of England.**—The Eighth Macloghlin Scholarship has been awarded to David Somerset Short, of Bristol Grammar School.

